# Pulmonary embolism diagnostics from the driver function

**DOI:** 10.1186/cc9438

**Published:** 2011-03-11

**Authors:** DJ Stevenson, J Revie, JG Chase, CE Hann, A Le Compte, GM Shaw, B Lambermont, P Kolh, T Desaive

**Affiliations:** 1 University of Canterbury, Christchurch, New Zealand; 2 Christchurch Hospital, Christchurch, New Zealand; 3 University of Liege, Belgium

## Introduction

Ventricular driver functions are not readily measured in the ICU, but can clearly indicate the development of pulmonary embolism (PE) otherwise difficult to diagnose. Recent work has developed accurate methods of measuring these driver functions from readily available ICU measurements. This research tests those methods by assessing the ability of these driver functions to diagnose the evolution of PE.

## Methods

PE was induced in five pigs with cardiac measurements taken every 30 minutes. Pig-specific driver functions are estimated at each time point from aortic artery pressure waveforms. Increases over time in two validated model-based metrics indicate PE: pulmonary artery resistance (Rpul); and the Right Ventricle Expansion Index (RVEI). Rpul and RVEI at each time point were paired to specific points on the right driver function that change as PE is induced. The significant points of interest are: (1) left-shoulder (LS) of the right driver function (correlated with the dead-space volume); (2) maximum pressure gradient (MPG) of the right driver function (related to compliance); and (3) the total area (TA) of the right driver function (analogous to work done by the ventricle). Correlations are calculated for each pig, and for measurements and driver functions averaged across all five pigs to see a general trend.

## Results

Pig-specific correlations were median (range): (1) RVEI to LS: 0.56 (range: 0.33 to 0.99); (2) RVEI to MPG: 0.59 (range: 0.25 to 0.99); and (3) Rpul to TA: 0.53 (range: 0.04 to 0.85). Correlation levels were not consistent across pigs or metrics with the maximum for each pig across the three metrics of (0.99, 0.85, 0.56, 0.54, 0.59), indicating inter-pig variability in the experimental response to PE and its impact on the identified driver functions. Averaging the data and driver functions over the five-pig cohort yielded excellent correlations between Rpul, RVEI and the estimated right driver function of: (1) RVEI to LS: *R *= 0.98, (2) RVEI to MPG: *R *= 0.98; and (3) Rpul to TA: *R *= 0.96. These results show the potential diagnostic capability of this approach in this limited animal trial.

## Conclusions

This research suggests that PE can be diagnosed and tracked from knowledge of a model-based driver function developed from readily available ICU measurements. Further animal and human validation is required to confirm these results.

